# Book Review: Modern Psychometrics With R

**DOI:** 10.3389/fpsyg.2020.00606

**Published:** 2020-04-15

**Authors:** Alexander Robitzsch

**Affiliations:** ^1^IPN – Leibniz Institute for Science and Mathematics Education, Kiel, Germany; ^2^Centre for International Student Assessment (ZIB), Kiel, Germany

**Keywords:** psychometrics, R, classical test theory, structural equating modeling, item response modeling

The book “Modern Psychometrics with R” by Patrick Mair provides extensive information about recent R (R Core Team, 2020) implementations of psychometric models. The book follows a broad definition of psychometrics in order to meet “the statistical needs of modern measurement” and exceeds the standard definition of psychological measurement (Mair, 2018, p. v). The author admits that several uncommon topics are included in the book, and the strength of the book is in its discussion of a broad range of R packages. The author has extensive expertise in the R community of psychometrics and is the maintainer of the CRAN task view “Psychometrics” (Mair, 2019). The book covers 14 chapters, and in the following review, I classify the chapters into core, broadened, and uncommon psychometrics and provide a discussion of the contents. In Table 1, the titles and the R packages used in the chapters are displayed.

**Table 1:** Table of contents and R packages used in Mair (2018).

**Chapter**	**Packages**
1. Classical test theory (15 pages)	**psych**^**c**^ (3), **lme4** (1), **gtheory** (189)
2. Factor analysis (46 pages)	**psych**^**c**^ (3), **lavaan**^**c**^ (15), **semTools** (30), **BayesFM** (93)
3. Path analysis and structural equation models (31 pages)	**lavaan**^**c**^ (15), **QuantPsych** (43), **mediation** (39), **nonnest2** (–)
4. Item response theory (65 pages)	**eRm**^**c**^ (64), **ltm**^**c**^ (45), **mirt**^**c**^ (31), **lordif** (97), **psychotree**^**c**^ (49), **Gifi** (179), **MCMCpack** (21)
5. Preference modeling (17 pages)	**BradleyTerry2** (17), **psychotree**^**c**^ (49), **BTLLasso** (96), **prefmod**^**c**^ (58)
6. Principal component analysis and extensions (32 pages)	**psych**^**c**^ (3), **elasticnet** (37), **multiway** (112), **eegkit** (124)
7. Correspondence analysis (20 pages)	**anacor**^**c**^ (91), **ca**^**c**^ (25), **cfa** (–)
8. Gifi methods (26 pages)	**Gifi** (179), **homals**^**c**^ (84), **aspect** (111)
9. Multidimensional scaling (31 pages)	**smacof**^**c**^ (57)
10. Biplots (23 pages)	**calibrate** (–), **bpca** (92), **smacof**^**c**^ (57), **anacor**^**c**^ (91)
11. Networks (22 pages)	**igraph** (–), **qgraph** (22), **network** (–), **latentnet** (–), **bnlearn** (–)
12. Parametric cluster analysis and mixture regression (30 pages)	**mclust** (–), **poLCA** (36), **flexmix** (5), **profdpm** (–), **tm** (–), **ldatuning** (–), **topicmodels** (–)
13. Modeling trajectories and time series (43 pages)	**depmixS4** (–), **forecast** (–), **strucchange** (–), **lmtest** (–), **tseries** (–), **fda** (–), **fda.usc** (–)
14. Analysis of fMRI data (42 pages)	**fmri** (–), **AnalyzeFMRI** (74), **ggBrain** (–), **brainR** (–), **RNiftyReg** (–), **class** (–), **multcomp** (–), **mmand** (–), **brainwaver** (–)

c *= R package contained in CRAN task view “Psychometrics” (CTVP; Mair, 2019). Numbers in parentheses after R packages are download ranks from the CRAN server in 2019 from the 221 R packages listed in the CTVP. The download statistics were obtained using the R package **cranlogs** (Csárdi, 2019). A hyphen (“–”) indicates that the respective package is not included in the CTVP list of R packages*.

## Core Psychometrics (Chapters 1–5; 174 pages)

In Chapter 1, the basics of classical test theory (CTT) and reliability measures are discussed, and the generalizability theory is also introduced. Chapter 2 provides an overview of exploratory and confirmatory factor analysis (CFA), as well as briefly discussing multiple-group and multilevel CFA models. Chapter 3 describes structural equation models (SEMs). Before introducing SEMs in their general form, the chapter starts with path models and (moderated) mediation models. In Chapter 4, unidimensional item response theory (IRT) models, as well as multidimensional exploratory and confirmatory IRT models, are described. The chapter also discusses differential item functioning techniques (i.e., logistic regression and tree methods). In Chapter 5, the analysis of pairwise comparisons and ranking data is considered. As an extension, in Chapters 2 to 5, longitudinal and Bayesian variants of CFA, SEM, and IRT models are discussed. In the first part of the book, the use of regularization techniques (e.g., employing the lasso penalty) is addressed only for the Bradley-Terry model in Chapter 5 (and later in Chapter 6) but could also be a valuable extension for CFA, SEM, and IRT models (see, for example, Jacobucci et al., 2016). It is my opinion that equating and linking techniques should also be recognized as part of core psychometrics (González and Wiberg, 2017).

## Broadened Psychometrics (Chapters 11–13; 95 pages)

In Chapter 11, network models in which the objects can be either persons or variables are discussed, and Bayesian networks are introduced. An extension to latent variable network models (Epskamp et al., 2018) would be helpful and could be considered in a future edition of the book. Chapter 12 explains model-based clustering approaches, mainly mixture regression, latent class analysis, and Dirichlet process clustering. Although mixture latent variable models are popular in applied research, they are not mentioned in this chapter. Chapter 13 discusses techniques for longitudinal data: hidden Markov models, single-subject time series, and functional data analysis. Readers could also find developments in multiple-subject (dynamic) time series models (mainly in the software M*plus*) and in continuous time models (Driver et al., 2017) interesting. In my view, broadened psychometrics should also include meta-analysis (Viechtbauer, 2010; Cheung, 2015).

## Uncommon Psychometrics (Chapters 6–10, Chapter 14; 174 Pages)

Chapter 6 starts with a detailed treatment of principal component analysis. It also introduces multiway analysis and independent component analysis. In Chapter 7, simple and multiple correspondence analysis and configural frequency analysis are explained. Chapter 8 focuses on a collection of exploratory techniques that are labeled as Gifi methods. Chapters 9 and 10 give an overview of multidimensional scaling and biplot techniques, respectively. Chapter 14 explains techniques for analyzing fMRI data that are seldom found in the psychometric research literature. The techniques described in Chapters 7 to 10 are also not frequently found in psychometric and applied research, maybe due to their mainly exploratory nature.

## Classical, Modern, and Future Psychometrics

To sum up, the book is a valuable and well-structured resource that forms links to a variety of R packages and, therefore, differs in content and depth from many other psychometrics books (e.g., Beaujean, 2014; Desjardins and Bulut, 2018; Paek and Cole, 2019). Given the variety of R packages used, I would have preferred the author to consistently use the package :: function notation to write the R function throughout the book^1^. In my opinion, a contemporary psychometrics book should put more emphasis on CTT (Nunnally and Bernstein, 1994; Meyer, 2010), CFA, and IRT models and I would like it to be more focused on design-based reliability (CTT) instead of model-based reliability (CFA and IRT models)^2^. The division of the book into core, broadened, and uncommon psychometrics makes it unique. However, this means that it cannot be the first choice for a companion book in an ordinary psychometrics course.

It is impressive how the R software has evolved in the last 10 years (see, for example, the **lavaan** and **mirt** packages; Chalmers, 2012; Rosseel, 2012). Given the rapid development of R packages, a new edition of this book would certainly be needed again in 10 years. In my opinion, R has made impressive progress, but R packages can still not fully compete with flexible and high-quality commercial software programs such as *LatentGold* or *Mplus*. The future will show whether equally flexible alternative open-source R packages have been developed. Maybe general-purpose software (like **BUGS** and its successors: Lunn et al., 2009; **OpenMx**: Neale et al., 2016; **Stan**: Carpenter et al., 2017; or **TMB**: Kristensen et al., 2016) might become more widespread in applied research in the future.

## Author Contributions

AR was responsible for the writing.

### Conflict of Interest

The author declares that the research was conducted in the absence of any commercial or financial relationships that could be construed as a potential conflict of interest.

## Footnotes

^1^If this notation is not used, function conflicts could occur if R functions with the same name occur in different (loaded) R packages. For example, Cronbach's alpha is available in several packages with a function name alpha (**psych**, **psychometric**, **coefficientalpha**, **plspm**, **epiDisplay**).Therefore, it makes sense to explicitly write psych::alpha if the function from the **psych** package is requested. Another critical example is the function name cfa that is used in the **lavaan** and **sem** package (for confirmatory factor analysis) as well as in the **cfa** package (for configural frequency analysis).

^2^In fact, as opposed what is frequently claimed in recent literature, CTT or generalizability theory have fewer assumptions than CFA or IRT because items are treated as random (i.e., exchangeable) in the former case while they are considered as fixed in the latter case (see Brennan, 2011).
